# Optimizing early identification and management of trastuzumab deruxtecan-related interstitial lung disease: insights from a multidisciplinary expert panel

**DOI:** 10.3389/fonc.2026.1899114

**Published:** 2026-10-14

**Authors:** Luca Licata, Camilla Lisanti, Grazia Arpino, Lorenzo Cereser, Nicholas Landini, Francesco Macagno, Francesco Pantano, Ida Paris, Mirco Pistelli, Paolo Tarsia, Giampaolo Bianchini

**Affiliations:** 1 Department of Medical Oncology, IRCCS San Raffaele Scientific Institute, Milan, Italy; 2 Department of Medical Oncology, Centro di Riferimento Oncologico di Aviano (CRO) IRCCS, Aviano, Italy; 3 Department of Clinical Medicine and Surgery, University of Naples "Federico II", Naples, Italy; 4 Sbarro Institute at Temple University Philadelphia, Philadelphia, PA, United States; 5 Department of Medicine (DMED), Institute of Radiology, University of Udine, Udine, Italy; 6 Institute of Radiology, University Hospital S. Maria della Misericordia, Azienda Sanitaria Universitaria Friuli Centrale (ASUFC), Udine, Italy; 7 Department of Radiological, Oncological and Pathological Sciences, Policlinico Umberto I Hospital, “Sapienza” Rome University, Rome, Italy; 8 Unit of Pneumology, Department of Neurosciences, Sensory Organs and Thorax, Fondazione Policlinico Universitario A. Gemelli IRCCS, Rome, Italy; 9 Department of Medical Oncology, Campus Bio-Medico University of Rome, Rome, Italy; 10 Department of Woman and Child Health, Fondazione Policlinico Universitario A. Gemelli IRCCS, Rome, Italy; 11 Department of Medical Oncology, Azienda Ospedaliera Universitaria delle Marche, Ancona, Italy; 12 Head of the Respiratory Unit, ASST Grande Ospedale Metropolitano Niguarda, Milan, Italy; 13 Vita-Salute San Raffaele University, Milan, Italy

**Keywords:** breast cancer, high-resolution computed tomography, interstitial lung disease, multidisciplinary management, trastuzumab deruxtecan

## Abstract

**Purpose:**

Trastuzumab deruxtecan (T-DXd) is a HER2-directed antibody–drug conjugate that has demonstrated substantial clinical efficacy across HER2-expressing malignancies. However, interstitial lung disease (ILD) represents a clinically relevant and potentially serious adverse event associated with its use, requiring early recognition, exclusion of alternative etiologies and coordinated multidisciplinary management. Delayed identification may increase the risk of severe pulmonary toxicity and premature discontinuation of an otherwise highly effective therapy. In this manuscript, we aim to provide practical guidance on the early detection and management of T-DXd-related ILD.

**Methods:**

A structured multidisciplinary discussion involving oncologists, pneumologists, and radiologists was conducted to evaluate the epidemiology, risk factors, diagnostic pathways, and management strategies for ILD in patients treated with T-DXd. Evidence from the published literature was reviewed to inform and support the panel’s recommendations.

**Results:**

ILD may arise in up to 15% of patients receiving T-DXd. While most events occur during the first year of treatment, continued vigilance is warranted throughout therapy. High-resolution computed tomography (HRCT) is essential for early detection, and prompt treatment interruption combined with timely corticosteroid therapy is critical to prevent progression. The panel emphasized the importance of standardized imaging protocols, structured radiologic reporting, early multidisciplinary involvement, and patient education to facilitate prompt recognition and management.

**Conclusion:**

Proactive surveillance strategies, including baseline and interval HRCT, multidisciplinary collaboration and patient-centered monitoring, are essential to minimize ILD-related morbidity while preserving access to T-DXd therapy. The implementation of standardized clinical pathways and the development of biomarker-driven approaches may further improve personalized ILD risk management.

## Introduction

Trastuzumab deruxtecan (T-DXd) is a HER2-directed antibody–drug conjugate that has significantly improved outcomes across multiple settings of HER2-expressing breast cancer. Initially developed for HER2-positive pretreated metastatic breast cancer, its therapeutic role has rapidly expanded to include HER2-low/ultralow tumors (1, 2), as well as early-stage (3, 4) and first-line HER2-positive metastatic disease (5).

The rapidly expanding indications and increasing use of T-DXd have substantially enlarged the population of patients exposed to this treatment. As a result, recognizing and managing T-DXd-related adverse events has become an increasingly important challenge in routine clinical practice. While the most common toxicities – including nausea and vomiting, fatigue, gastrointestinal, and hematologic adverse events – are generally manageable with appropriate supportive care (6–8), interstitial lung disease (ILD) remains the most clinically significant toxicity because of its potential severity, the risk of treatment interruption or permanent discontinuation, and the possibility of fatal outcomes.

This evolving therapeutic landscape calls for a paradigm shift toward proactive, multidisciplinary surveillance and toxicity management (9).

ILD is an umbrella term encompassing a spectrum of interstitial lung abnormalities that may progress to irreversible pulmonary fibrosis (10). In patients receiving T-DXd, establishing the diagnosis is often challenging because ILD remains a diagnosis of exclusion. Therefore, alternative causes of respiratory symptoms and radiologic abnormalities –including infection, cardiogenic pulmonary edema, radiation-induced lung injury, and disease progression– must be carefully excluded before attributing these findings to T-DXd-related ILD (11, 12). The reported incidence of T-DXd-related ILD varies across clinical trials, reflecting differences in patient populations, baseline risk factors, disease settings and duration of follow-up (1–6, 13, 14). Across studies, most events were grade 1–2, whereas severe and fatal events remained uncommon but clinically relevant (15–17). Although most cases are manageable if detected early, higher-grade ILD events can lead to significant morbidity and require permanent treatment discontinuation when grade ≥2 (8, 11). Reported risk factors include older age (>65 years), pre-existing lung comorbidities, Asian race, impaired renal function, >4 years since diagnosis, smoking history, baseline lung metastases and a baseline oxygenation <95% (11, 18). Additionally, real-world evidence suggested prior immunotherapy as a potential risk factor for ILD in patients receiving T-DXd (19).

The diagnosis of T-DXd-related ILD can be challenging, as symptoms such as dyspnea, cough and fever are non-specific and may overlap with infection or disease progression. Furthermore, some patients are asymptomatic at onset, reinforcing the importance of systematic radiologic surveillance (20). High-resolution computed tomography (HRCT), performed with thin sections and dedicated high-resolution reconstructions of the lung parenchyma, is the most sensitive imaging modality for detecting early manifestations of drug-induced lung injury (21). However, limited access in some centers may hinder the routine use of contrast-enhanced imaging at strictly short intervals. In routine practice, non-contrast HRCT every 10–12 weeks can be considered adequate for standard-risk patients and may offer practical advantages, including shorter scan times, lower resource utilization and reduced waiting times. Multidisciplinary collaboration among oncologists, radiologists, and pneumologists is essential to optimize patient outcomes. Clinical guidelines and expert consensus also emphasize patient education on early symptom recognition and structured imaging protocols to reduce the risk of severe ILD (21).

Despite increasing awareness, the absence of standardized ILD monitoring pathways has resulted in variability in diagnostic approaches and management strategies across centers.

In October 2024, a multidisciplinary group of experts from 10 Hospitals in Italy convened to discuss the optimal management of T-DXd-related ILD, aiming to develop practical recommendations tailored to the Italian context. Here, we review available evidence and report the conclusions of this multidisciplinary board.

## Materials and methods

The multidisciplinary advisory board comprised the co-authors of this manuscript, including medical oncologists, pulmonologists and thoracic radiologists with recognized expertise in breast cancer, T-DXd treatment, and diagnosis and management of drug-related ILD. Panel members were selected based on their clinical experience, multidisciplinary expertise and active involvement in the management of patients receiving T-DXd across Italian referral centers.

The primary aim of the meeting was to identify current challenges in the early recognition and management of T-DXd-related ILD, in order to develop practical recommendations applicable to routine clinical practice. Recommendations were developed through structured multidisciplinary discussions, open scientific debate and critical appraisal of the available literature. Consensus was reached through discussion until agreement was achieved among all participants, and the resulting recommendations represent the shared expert opinion of the panel, informed by current evidence and real-world clinical experience. This manuscript represents an expert panel review and was not based on a systematic literature search. Recommendations for which prospective evidence remains limited should therefore be interpreted as expert consensus informed by available evidence and current international guidance.

## Clinical relevance of T-DXd-related ILD

The occurrence of ILD may compromise treatment continuity and therapeutic sequencing. Current guidance recommends immediate interruption at the first suspicion of ILD and permanent discontinuation of T-DXd for grade ≥2 events (21, 22). Although necessary for patient safety, these measures may limit exposure to a highly effective therapy.

Conversely, early detection of grade 1 events followed by prompt management may prevent progression to higher grades and preserve the option of T-DXd rechallenge in selected patients (23, 24). Therefore, ILD surveillance is not only a safety measure but also a strategy to maintain oncologic benefit while limiting pulmonary toxicity.

The panel emphasized the dual clinical relevance of T-DXd-related ILD: a potentially life-threatening toxicity and a modifiable determinant of treatment continuity. Early radiologic detection – ideally before symptom onset – should be integral to therapeutic strategy rather than a reactive measure (12, 23).

From a practical perspective, three priorities emerge:

1. Proactive surveillance, particularly important during the first 6–12 months of therapy, when the incidence of ILD is highest. However, because ILD may also develop later during treatment, continued clinical vigilance and appropriate imaging surveillance should be maintained throughout the entire treatment course (5, 11, 13, 14, 25).
2. Immediate interruption at the first radiologic suspicion, even in asymptomatic patients, to prevent progression to grade ≥2 events (22, 23).
3. Individualized risk–benefit assessment when considering rechallenge after resolution of grade 1 ILD, balancing oncologic urgency with pulmonary risk and ensuring close monitoring (24, 26).

Preserving the clinical benefit of T-DXd ultimately requires structured monitoring pathways that mitigate toxicity without unnecessary loss of therapeutic opportunity (26, 27).

## Toward standardized clinical-radiological pathways for T-DXd-related ILD

Clinical practice remains heterogeneous across oncology centers in terms of imaging protocols, surveillance timing, radiologic interpretation and referral to pneumology.

Radiologic reporting is also not standardized: terminology describing lung changes varies across centers and readers, potentially delaying recognition of early drug-related patterns such as organizing pneumonia, particularly when subtle findings are present (28). The lack of structured reporting may further impair timely communication between oncologists and radiologists.

Real-world data suggest that reported ILD incidence and severity may also depend on surveillance intensity and diagnostic practices (19, 27).

Beyond imaging variability, a key limitation is the lack of shared diagnostic pathways for suspected ILD. International guidelines define drug-related ILD as a diagnosis of exclusion requiring integration of clinical, radiologic and laboratory data (21, 22), yet real-world workflows for differential diagnosis vary substantially across institutions.

Recent expert recommendations stressed the need to harmonize baseline assessment, imaging intervals, radiologic terminology and multidisciplinary involvement (26), aligning national practice with international guidance (22, 23).

An effective shared framework should include mandatory baseline HRCT before treatment initiation; uniform imaging intervals, particularly during the first year when ILD incidence is highest, while maintaining continued clinical and radiologic vigilance throughout the entire treatment course; standardized ILD reporting terminology; clear criteria for pneumology referral and agreed algorithms for treatment interruption, corticosteroid use and rechallenge.

Three key actions are proposed:

1. Implementation of structured imaging requests, clearly indicating that the patient is receiving T-DXd, specifying HRCT when appropriate, and including the date of treatment initiation, the number of treatment cycles, previous potentially pneumotoxic anticancer therapies (including thoracic radiotherapy), and the clinical indication for the examination, in order to enhance radiologic vigilance and consistency (28).
2. Early multidisciplinary involvement, with pulmonology consultation at the first radiologic suspicion (23, 26).
3. Adoption of a shared national algorithm integrating surveillance timing, grade-based management, and rechallenge criteria to ensure consistent decision-making across institutions.

The panel stressed that harmonization does not imply rigid standardization, but rather the establishment of a common minimum framework within which individualized clinical judgment can operate.

## A patient-centered workflow for T-DXd-related ILD

A patient-centered approach to T-DXd-related ILD requires baseline risk stratification, structured monitoring, multidisciplinary coordination, and active patient engagement.

### Risk assessment, patient selection and education

The pretreatment visit before T-DXd initiation is critical for structured risk assessment and patient education. Baseline evaluation should include a systematic review of known ILD risk factors and definition of corresponding surveillance strategies (**Table 1**).

**Table 1:** Clinical risk factors for T-DXd-related ILD and implications for surveillance strategies.

Risk factor	Clinical rationale	Suggested practical implication
Age >65 years	Increased susceptibility to drug-induced pulmonary toxicity	Closer clinical assessment and radiologic surveillance, particularly during the first year of treatment
Pre-existing ILD or chronic lung disease	Reduced pulmonary reserve and higher risk of ILD development	Baseline pneumology evaluation and careful comparison with baseline HRCT before attributing new findings to T-DXd
Asian ethnicity	Higher incidence reported in pooled analyses	Increased awareness and closer monitoring throughout treatment
Moderate/severe renal impairment	Associated with increased ILD risk in pooled analyses	Consider closer imaging follow-up and multidisciplinary assessment
Baseline oxygen saturation <95%	May identify patients with limited respiratory reserve	Careful clinical follow-up and prompt evaluation of new respiratory symptoms
Smoking history	May complicate the interpretation of respiratory symptoms and CT findings	Reinforce patient education and maintain a low threshold for diagnostic work-up
Lung metastases	May complicate the differential diagnosis of new pulmonary abnormalities	Careful comparison with baseline imaging and multidisciplinary radiologic review
>4 years since initial diagnosis	Associated with increased ILD incidence in pooled analyses	Maintain heightened awareness during long-term treatment

Risk factors are derived primarily from pooled analyses of T-DXd clinical trials (11) and should be considered collectively rather than individually when planning patient surveillance.

HRCT, high-resolution computed tomography; ILD, interstitial lung disease; T-DXd, trastuzumab deruxtecan.

Although no validated predictive score exists, identifying pulmonary vulnerability may guide monitoring intensity and multidisciplinary involvement.

Patients with pre-existing lung disease or reduced respiratory reserve require particular caution. Frailty, poor performance status and polypharmacy may further complicate ILD recognition and management, reinforcing the need for individualized therapeutic planning.

Patients with brain metastases represent a particularly vulnerable subgroup, as high-dose or prolonged corticosteroids may mask early respiratory symptoms and delay ILD recognition. Fatal ILD events have been reported in this population, including patients receiving corticosteroids for central nervous system disease (1, 26). In this context, differential diagnosis can be challenging, as neurologic symptoms can overlap with respiratory manifestations or opportunistic infections, particularly during prolonged steroid exposure. In selected patients receiving extended corticosteroid therapy, antimicrobial prophylaxis (e.g., trimethoprim–sulfamethoxazole) may be considered according to expert recommendations (26).

Transparent communication about ILD risk is recommended to support adherence to monitoring and enable timely intervention (6, 22). Patient education through structured counseling, written materials and, where feasible, home pulse oximetry may complement individualized surveillance strategies. Shared decision-making should explicitly address the balance between oncologic benefit and pulmonary risk, particularly in frail patients or those with comorbidities.

### Baseline evaluation before T-DXd initiation

HRCT is the gold standard imaging modality for the detection and assessment of T-DXd-related ILD. For the purpose of ILD surveillance, HRCT refers to a thin-section chest CT with dedicated high-resolution lung parenchymal reconstruction, preferably performed without contrast when the primary objective is parenchymal evaluation. By contrast, standard contrast-enhanced oncologic staging CT examinations, although appropriate for tumor assessment, may not always include thin-section acquisitions or dedicated high-resolution reconstruction algorithms, potentially reducing the sensitivity for detecting subtle interstitial abnormalities. Therefore, the imaging protocol should be selected according to the clinical indication and local availability (23). Imaging requests should explicitly indicate that the patient is receiving T-DXd and include relevant clinical information (such as the date of treatment initiation, the number of treatment cycles, previous potentially pneumotoxic anticancer therapies, particularly thoracic radiotherapy, and the clinical indication for the examination), to facilitate radiologic interpretation and early recognition of drug-related ILD, in accordance with current radiologic reporting recommendations (28).

Baseline pulmonary function testing, including spirometry and diffusion capacity for carbon monoxide, is not routinely required for all patients but may provide complementary functional information in patients with pre-existing lung disease (23); longitudinal functional trends may further aid interpretation (12). Accurate documentation of pre-existing interstitial alterations is essential to distinguish new drug-related changes from chronic findings, as well as direct comparison with baseline imaging is strongly recommended when ILD is suspected (22). In patients with significant pulmonary comorbidities or borderline findings, early pulmonologists’ involvement may support individualized surveillance and interpretation of equivocal radiologic changes.

### Structured monitoring and surveillance during the treatment phase

Routine surveillance should include systematic assessment of respiratory symptoms, physical examination (including chest examination), and measurement of oxygen saturation by pulse oximetry at each patient visit. Although no prospective studies have established an optimal HRCT surveillance schedule, for standard-risk patients the expert panel suggests baseline HRCT followed by imaging approximately every 10–12 weeks during the first year of treatment. This recommendation is based on expert consensus and current international guidance. Notably, disease assessments in the DESTINY clinical trials were generally performed every 6 weeks using standard CT, allowing frequent evaluation for potential pulmonary abnormalities, although dedicated HRCT was not mandated in those trials. While the incidence of ILD is highest during the first 12 months of therapy (11), continued clinical and radiologic vigilance should be maintained throughout treatment because late-onset ILD events have also been reported (5, 13, 23, 25). Higher-risk patients, such as those with brain metastases receiving prolonged corticosteroid therapy, may require closer surveillance (approximately every 8 weeks).

Radiologic reports should describe alterations (e.g., ground-glass opacities, organizing pneumonia) and their distribution, compare findings with baseline imaging, and explicitly comment on compatibility with drug-related ILD (12, 28). The multidisciplinary workflow for HRCT prescription, acquisition, interpretation and clinical communication during T-DXd treatment is illustrated in **Figure 1**.

**Figure 1:**
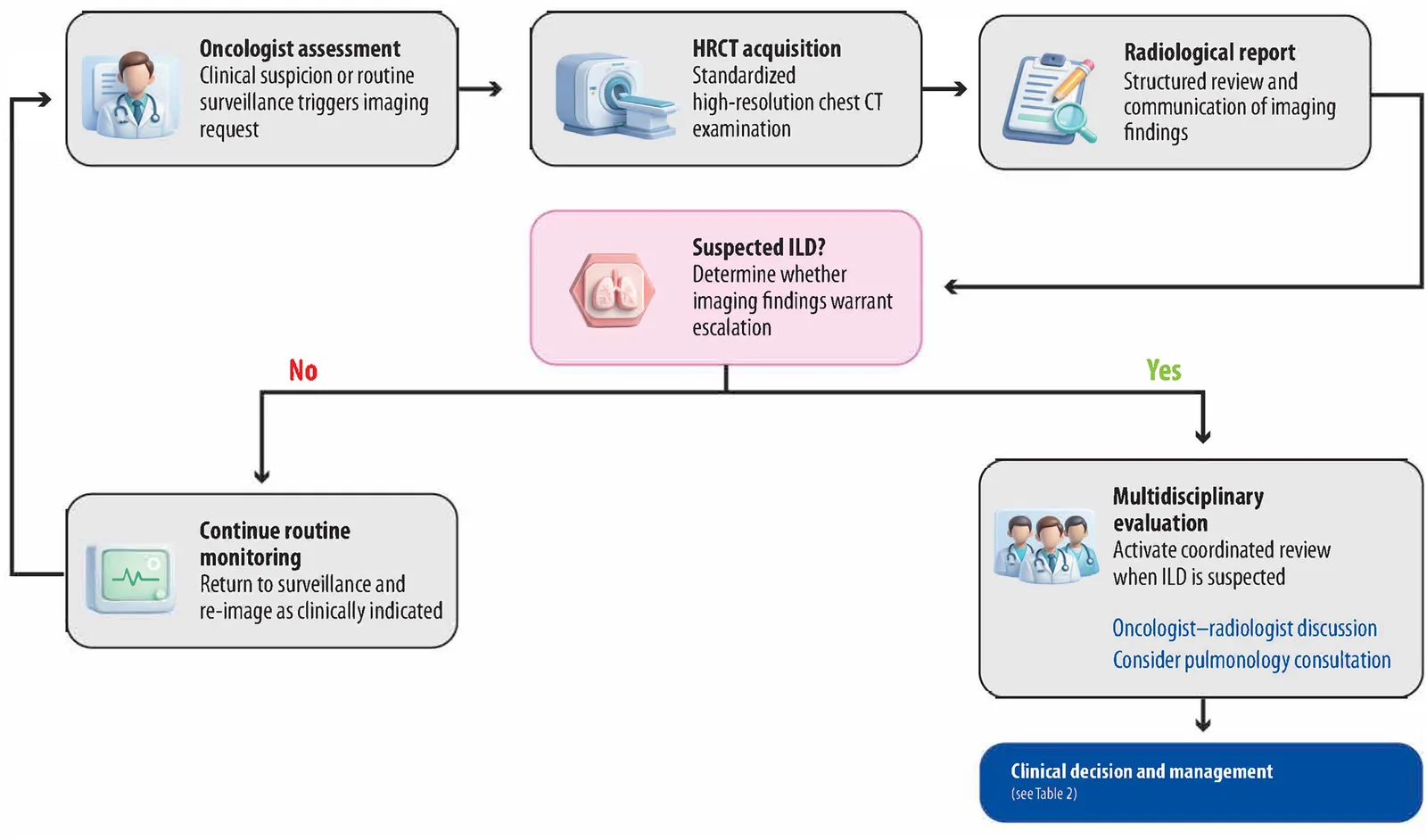
Multidisciplinary imaging workflow for early detection of T-DXd-related interstitial lung disease. Early identification of ILD requires coordinated interaction between oncologists and radiologists, including appropriate HRCT prescription, standardized reporting and direct communication when suspicious findings are detected. Arrows indicate the sequential flow from imaging prescription and acquisition to radiologic interpretation and communication of suspicious findings to the treating team. HRCT, high-resolution computed tomography; ILD, interstitial lung disease; T-DXd, trastuzumab deruxtecan.

### Differential diagnosis of ILD

The diagnosis of T-DXd-related ILD remains one of exclusion and requires systematic differential evaluation integrating clinical presentation, imaging findings, laboratory data and treatment history (21, 22).

Infection is one of the most frequent alternative diagnoses. Bacterial, viral (including cytomegalovirus), fungal (e.g., Pneumocystis jirovecii, Aspergillus spp.) and less commonly protozoal infections should be considered, particularly in immunocompromised patients or those receiving prolonged corticosteroids (26). Infection and drug-induced ILD may also coexist. Clinical context, inflammatory markers, microbiological testing, and – when indicated – bronchoalveolar lavage may aid clarification.

Progressive pulmonary metastases or lymphangitic carcinomatosis may mimic ILD and require careful comparison with baseline imaging and assessment of systemic disease status (12).

Prior exposure to pneumotoxic agents (e.g., CDK4/6 inhibitors, everolimus, thoracic radiotherapy) further complicates attribution (19). Radiation-induced pneumonitis or fibrosis should be distinguished by correlating abnormalities with radiation fields and the period of radiotherapy.

Cardiogenic pulmonary edema, diffuse alveolar hemorrhage and infectious pneumonia may present with overlapping radiologic findings too (29). Clinical assessment, echocardiography, laboratory parameters, including BNP/NT-proBNP, hemoglobin levels and inflammatory markers, may assist in the differential diagnosis. In patients receiving corticosteroids for brain metastases, early respiratory symptoms may be attenuated and steroid-induced immunosuppression increases susceptibility to opportunistic infections that can radiologically resemble ILD (26). Diagnostic vigilance and prompt multidisciplinary evaluation are therefore essential in this subgroup.

Suspected ILD should prompt immediate multidisciplinary discussion, as a coordinated, parallel evaluation across specialties may reduce delays compared with sequential referral pathways. Practical recommendations include early radiology and pulmonology involvement at first suspicion, parallel infection work-up and a low threshold for treatment interruption while differential diagnosis is ongoing.

### ILD management and treatment decision-making

Management of T-DXd-related ILD is guided by toxicity grade, clinical presentation and radiologic extent, in accordance with international guidelines and product recommendations (**Table 2**) (20–22).

**Table 2:** Practical multidisciplinary management of T-DXd-related ILD according to Common Terminology Criteria for Adverse Events (CTCAE) grade.

CTCAE grade	T-DXd management	Corticosteroid therapy & multidisciplinary management	Follow-up imaging	Rechallenge
Grade 1 (asymptomatic)	Temporarily withhold T-DXd	Consider corticosteroids based on the extent of radiologic abnormalities and clinical judgment. Multidisciplinary discussion recommended.	Repeat HRCT after 2weeks, then according to clinical evolution until complete radiologic resolution	May be considered only after complete resolution of Grade 1 ILD, following multidisciplinary discussion, patient counseling, and careful risk–benefit assessment
Grade 2 (symptomatic)	Permanently discontinue T-DXd	Prompt initiation of systemic corticosteroids. Pneumology consultation recommended.	Repeat HRCT after 2 weeks to document response to treatment and until complete radiologic resolution	Not recommended
Grade 3–4 (severe or life-threatening)	Permanently discontinue T-DXd	Hospitalization, high-dose systemic corticosteroids, and additional immunosuppressive therapy if clinically indicated. Intensive multidisciplinary management.	Serial HRCT according to clinical evolution until stabilization or complete resolution	Not recommended

Recommendations regarding follow-up imaging intervals and T-DXd rechallenge are based on the current prescribing information, international guidance, and expert panel consensus, as prospective evidence specifically addressing these aspects remains limited. CTCAE, Common Terminology Criteria for Adverse Events; HRCT, high-resolution computed tomography; ILD, interstitial lung disease; T-DXd, trastuzumab deruxtecan.

For grade 1 events (asymptomatic, radiologic findings only), T-DXd should be temporarily withheld. Corticosteroids may be considered based on the extent of radiologic abnormalities and their evolution, as well as clinical judgment. HRCT should be repeated after approximately 2 weeks and subsequently according to clinical evolution until complete radiologic resolution (20–22).

For grade ≥2 events (symptomatic), permanent discontinuation of T-DXd and prompt initiation of systemic corticosteroids are required. In more severe cases, hospitalization with respiratory support and high-dose intravenous corticosteroids may be necessary (20–22).

Systemic corticosteroids represent the cornerstone of ILD management. Early initiation in symptomatic patients is recommended to reduce the risk of progression and severe pulmonary impairment. Tapering should be individualized according to clinical and radiologic response.

Pulmonology consultation is warranted at the first radiologic suspicion or symptom onset. It should be strongly considered in grade ≥1 events and recommended in grade ≥2 events, as well as in cases of ambiguous radiologic patterns, lack of improvement despite corticosteroid therapy, and/or in patients with significant baseline pulmonary disease.

T-DXd rechallenge may be considered only in carefully selected patients with complete resolution of grade 1 ILD, following multidisciplinary discussion and transparent communication regarding recurrence risk and therapeutic alternatives. Pooled analyses suggest retreatment can be feasible under strict monitoring, although recurrence risk remains (24). Rechallenge is not recommended after grade ≥2 ILD events.

Therapeutic decisions should balance oncologic urgency with pulmonary risk. In patients with high unmet oncologic need and limited alternatives, individualized multidisciplinary discussion may support carefully monitored management within current guideline and prescribing information constraints (26).

The overall multidisciplinary diagnostic and therapeutic pathway proposed by the expert panel is summarized in **Figure 2**, providing a practical framework for the prevention, early recognition, differential diagnosis and grade-adapted management of T-DXd-related ILD.

**Figure 2:**
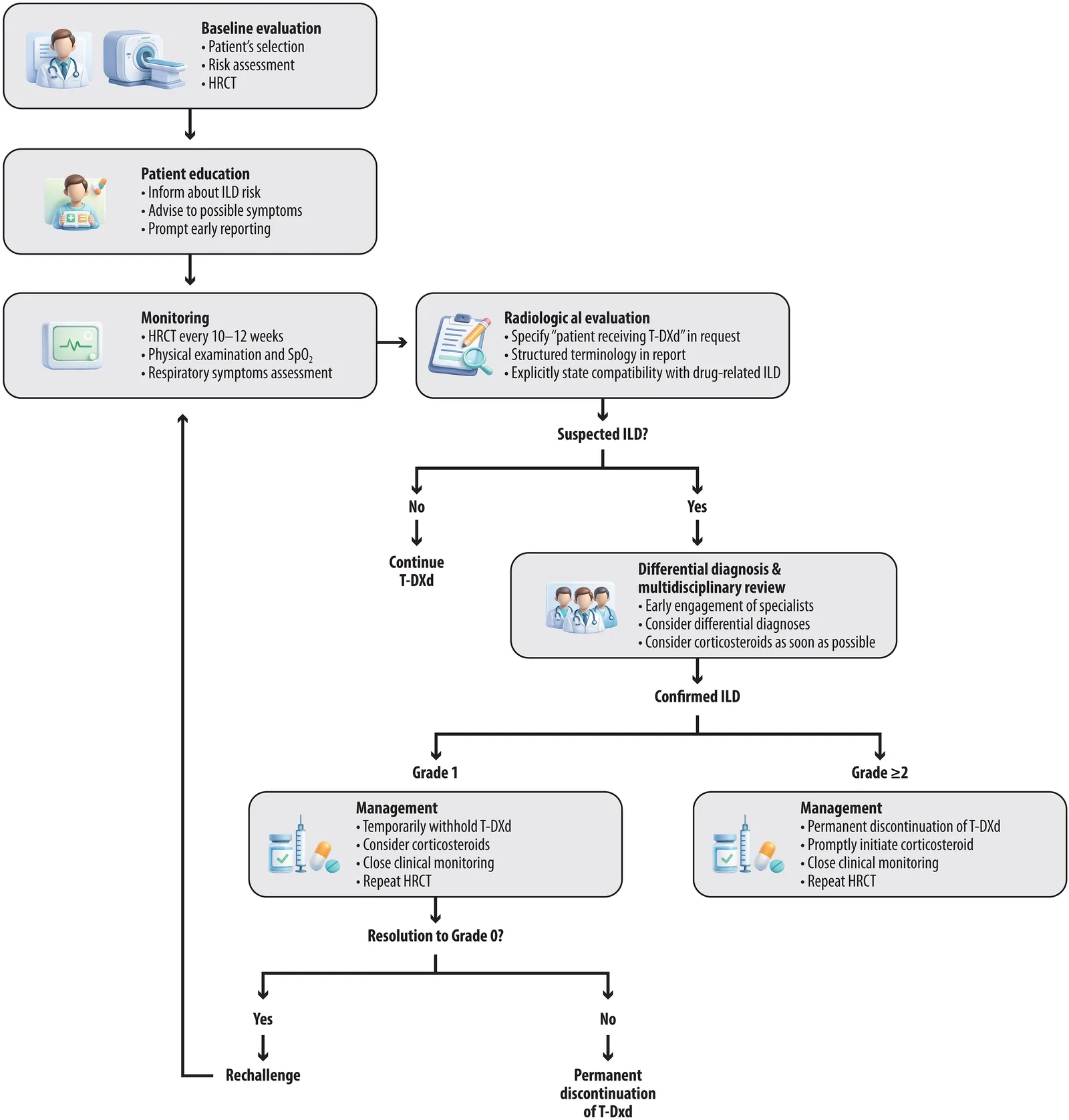
Standardized multidisciplinary algorithm for the prevention, diagnosis, and management of T-DXd-related interstitial lung disease. Arrows indicate the sequential clinical pathway from baseline assessment and surveillance to evaluation of suspected ILD and subsequent grade-adapted management. HRCT, high-resolution computed tomography; ILD, interstitial lung disease; T-DXd, trastuzumab deruxtecan.

## Future perspectives

Despite advances in structured monitoring, ILD associated with T-DXd remains only partially predictable. As the clinical use of T-DXd continues to expand across both early-stage and advanced breast cancer (3–5), the implementation of standardized ILD surveillance pathways will become increasingly important. Key research priorities include the identification of susceptibility biomarkers, refinement of risk stratification models integrating clinical and radiologic variables, optimization of surveillance intervals according to individual risk profiles, and better characterization of safe rechallenge strategies.

Emerging technologies may further enhance these efforts. Artificial intelligence-assisted interpretation of chest CT and radiomics-based imaging biomarkers have the potential to identify subtle imaging abnormalities, improve risk stratification, and support earlier recognition of drug-induced lung injury before clinically overt toxicity develops (30, 31). Although these technologies require prospective validation in patients receiving T-DXd, they represent promising strategies to further personalize surveillance and optimize multidisciplinary management. In parallel, digital health solutions, including electronic patient-reported outcomes and remote symptom monitoring, may facilitate earlier identification of new or worsening respiratory symptoms, particularly in patients requiring closer surveillance because of baseline risk factors (32, 33).

Translational studies exploring the biological mechanisms underlying T-DXd-related ILD may further clarify the contributions of cytotoxic injury, inflammatory pathways, and host-specific susceptibility (15, 34). Until validated predictive tools become available, rigorous monitoring, patient education, and close multidisciplinary coordination remain essential components of safe clinical practice. Proactive ILD management should, therefore, be regarded as an integral component of T-DXd therapy rather than a secondary safety measure.

## Conclusions

T-DXd has reshaped the therapeutic paradigm of HER2-expressing breast cancer, with its clinical use rapidly expanding across both metastatic and early-stage disease settings (1–5). However, ILD remains a clinically relevant and potentially life-threatening adverse event that may compromise treatment continuity and outcomes (11, 22).

Beyond its incidence, the clinical significance of T-DXd-related ILD lies in its impact on therapeutic strategy. Delayed recognition may result in severe pulmonary injury or premature discontinuation of an otherwise highly effective therapy, whereas early detection of low-grade events enables timely intervention and may preserve future treatment opportunities.

A structured framework – integrating baseline risk assessment, standardized HRCT surveillance, early multidisciplinary involvement, and grade-adapted management – may improve consistency of care and reduce the risk of severe events. In this context, three overarching principles should guide clinical practice: standardization to enhance patient safety; early multidisciplinary involvement at the first suspicion of ILD; and integration of algorithm-based strategies with individualized clinical judgment, particularly in complex cases or potential rechallenge scenarios.

Ultimately, timely recognition and coordinated management are essential to safely maximize the clinical benefit of T-DXd.
